# TTB Protects Astrocytes Against Oxygen-Glucose Deprivation/Reoxygenation-Induced Injury *via* Activation of Nrf2/HO-1 Signaling Pathway

**DOI:** 10.3389/fphar.2019.00792

**Published:** 2019-07-16

**Authors:** Liang Liu, Zhichen Zhao, Qimeng Yin, Xiaolu Zhang

**Affiliations:** ^1^Institute of Translational Medicine, Medical College, Yangzhou University, Yangzhou, China; ^2^Jiangsu Key Laboratory of Experimental & Translational Non-coding RNA Research, Yangzhou University, Yangzhou, China; ^3^Jiangsu Key Laboratory of Zoonosis, Jiangsu Co-innovation Center for Prevention and Control of Important Animal Infectious Diseases and Zoonoses, College of Veterinary Medicine, Yangzhou University, Yangzhou, China; ^4^Department of Pharmacy, Clinical Medical College, Yangzhou University, Yangzhou, China

**Keywords:** neonatal hypoxia/ischemic encephalopathy, TTB, oxygen-glucose deprivation/reoxygenation, astrocytes, Nrf2/HO-1 signaling pathway, HIF-1α, VEGF

## Abstract

Neonatal hypoxic/ischemic encephalopathy (NHIE) is a severe condition that leads to death or neurological disability in newborns. The underlying pathological mechanisms are unclear, and developing the target neuroprotective strategies are urgent. 2,7,2′-trihydroxy-4,4′7′-trimethoxy-1,1′-biphenanthrene (TTB) is a natural product isolated from *Cremastra appendiculata* (D. Don) Makino and *Liparis nervosa* (Thunb.) Lindl. TTB has demonstrated potent cytotoxic activity against stomach (HGC-27) and colon (HT-29) cancer cell lines. However, none of the studies have addressed the effects of TTB in NHIE. In the present study, an oxygen-glucose deprivation/reoxygenation (OGD/R)-induced astrocyte injury model was established to investigate the effect of TTB and its potential mechanisms. Our results showed that TTB alleviated the OGD/R-induced reactive oxygen species increase and the intracellular antioxidant capacity of superoxide dismutase activity decrease. Moreover, TTB potentially prolonged the activation state of the nuclear factor erythroid 2-related factor 2 (Nrf2)/heme oxygenase-1 (HO-1) pathway and maintained the protection against oxidative stress in OGD/R-induced astrocytes by inducing the nuclear translocation and up-regulation of Nrf2 along with the enhanced expression of the downstream target gene HO-1. Furthermore, TTB treatment diminished the accumulation of hypoxia-inducible factor-1α (HIF-1α) and vascular endothelial growth factor (VEGF) expression induced by OGD/R. We also found TTB-treated astrocytes reversed the inhibition of OGD/R on neurite growth of neurons by the astrocyte-neuron coculture system. In conclusion, TTB inhibited the OGD/R-induced astrocyte oxidative stress at least partially through the inhibition of HIF-1α and VEGF *via* the Nrf2/HO-1 signaling pathway.

## Introduction

Neonatal hypoxia/ischemic encephalopathy (NHIE), also described as stroke in the neonatal period, is one of the most prevalent causes of a potentially devastating neonatal brain injury with long-term neurological deficits such as mental retardation, cerebral palsy, motor deficits, epilepsy, and learning and behavioral disabilities, which affects 1 to 8 of every 1000 live term births, with the highest rates in developing countries (Dilenge et al., 2001; Kurinczuk et al., 2010). The cases of infantile cerebral palsy are caused by the same factors that cause adult cerebral palsy (Nelson, 2007). At present, therapeutic hypothermia protocols are formally endorsed treatments, which significantly improve outcomes by leading to delayed cell death. However, its effectiveness is limited in severe cases, as 40% to 50% of children with NHIE still die or suffer from long-term neurological disorders (Edwards et al., 2010). There are no effective pharmacological interventions available. To reduce the neurological consequences of NHIE, new and effective neuroprotective strategies are urgently needed.

Astrocytes are the largest population of glial cells in the brain and have been implicated in many functions as key mediators in the central nervous system (CNS). Astrocytes are highly involved in neuronal migration, adaptive plasticity, and synaptogenesis in the developing brain (Ullian et al., 2001; Guizzetti et al., 2014). The developing neonatal brain is particularly vulnerable to oxidative stress based on the immature free radical scavenging systems (Zorec et al., 2018). Several evidences have identified that NHIE causes long-lasting oxidative stress, a process aggravated by mitochondrial dysfunction. Reactive oxygen species (ROS) have been involved in the pathogenesis of NHIE and induce cell death *via* the oxidation of membrane lipids and proteins (Fatemi et al., 2009). Recently, a study demonstrated that astrocytes are a major source of increased brain ROS production during neonatal asphyxia (Parfenova et al., 2018).

Nuclear factor erythroid 2-related factor 2 (Nrf2) is a member of the basic region/leucine zipper transcription factor family that regulates several antioxidant pathways (Sandberg et al., 2014). Under unstressed conditions, Nrf2 is binding to the homodimeric protein Kelch-like ECH-associated protein 1 (Keap1), which becomes the Nrf2 Keap1 complex in the cytoplasm. In pathological processes, such as oxidative stress and other insult attacks, Nrf2 is activated by release from the antioxidant response element of Keap1 and translocated to the nucleus from the cytoplasm, which leads to accumulate in the nucleus, regulates genetic activities, and induces cytoprotective action (Cao et al., 2015). Nrf2 activation drives several functions, including antioxidative stress, antiapoptosis, and anti-inflammation, *via* several molecules and pathways (Shu et al., 2016).

2,7,2′-trihydroxy-4,4′7′-trimethoxy-1,1′-biphenanthrene (TTB) is a biphenanthrene isolated from *Cremastra appendiculata* (D. Don) Makino (Xue et al., 2006; Liu et al., 2016a) and *Liparis nervosa* (Thunb.) Lindl. (Liu et al., 2016b), which both belong to the family Orchidaceae. Research concerning bioactivities of TTB was very limited, and it was only reported to have cytotoxic activity against stomach (HGC-27) and colon (HT-29) cancer cell lines (Liu et al., 2016b). Therefore, it is necessary to explore other bioactivities of TTB.

Oxygen-glucose deprivation/reoxygenation (OGD/R) is a widely used cell model to mimic the aspects of cell death observed in a hypoxia brain injury model, including neonate HI and adult ischemic stroke (Cengiz et al., 2014; Tasca et al., 2015). Several recent studies demonstrated that Nrf2 was regulated by special compounds in the rat neonatal HI brain injury model (Cui et al., 2017; Gao et al., 2018). We speculated that TTB may offer neuroprotection in part by regulating Nrf2 in reactive astrocytes. In the current study, we investigated the effect of TTB on the OGD/R-induced astrocyte injury model, which is to mimic NHIE *in vitro*. We observed that Nrf2 activation *via* TTB treatment improved astrocyte function by targeting oxidative stress. Our findings suggested that astrocytic Nrf2 could be a potential therapeutic target for the treatment of NHIE.

## Materials and Methods

### Compound

The ethyl acetate extract of *L. nervosa* (Thunb.) Lindl. was isolated and purified using repeated column chromatography over Sephadex LH-20, RP-C18, silica gel, and semi-preparative high performance liquid chromatography (HPLC) to obtain TTB. The purity of TTB was at least 99% as judged by HPLC analysis. All the extraction, separation, and purification were performed by our group (Liu et al., 2016b).

### Cell Culture

Postnatal day 1 Sprague–Dawley rats were purchased from the Comparative Medicine Center of Yangzhou University (Yangzhou, China) and used for culturing astrocytes as described previously (Hertz et al., 1998; Zhang et al., 2014). Briefly, the cerebral cortex was taken in a sterile environment and then dispersed with 0.25% trypsin (Gibco Co., Grand Island, NY, USA) for 10 min at 37°C. The cells were plated in 75 cm^2^ flasks precoated with 40 µg/ml poly-d-lysine, grown in high-glucose Dulbecco’s modified Eagle medium (DMEM; Gibco Co.) containing 10% fetal bovine serum (FBS; Gibco Co)., 100 units/ml penicillin, and 100 μg/ml streptomycin (Solarbio, Beijing, China), and placed in an incubator at 5% CO_2_, 95% air at 37°C. The flasks were gentle shaken about 150 times by hand to remove the layer of nonadherent cells growing on the top of the flat monolayer when changing the medium every 2 to 3 days. More than 95% astrocytes were achieved by the cultures. After 14 days in culture, astrocytes were grown to confluence and then plated in the appropriate vessel. When the cultures reached 70% to 80% confluence, cells were ready for treatment.

### Establishment of OGD/R-Induced Injury of Astrocytes

The OGD/R model was established in astrocytes. Briefly, the cells were washed twice with phosphate-buffered saline (PBS) and incubated in glucose- and FBS-free medium and then placed in an anoxic incubator at 94% N_2_, 1% O_2_, 5% CO_2_ at 37°C. After OGD for 6 h, the medium was changed back to high-glucose DMEM containing 10% FBS and returned to the normal oxygen incubator for another 24 h. The blank control group in the experiment was always kept in a normal oxygen incubator and cultured in high-glucose medium containing 10% FBS. The cells were given OGD/R treatment with or without TTB (1.5625, 6.25, and 25 µM). For certain experiments, the astrocytes were preincubated in ML385 (Nrf2-specific inhibitor) for 12 h before OGD/R.

### Cell Viability

Primary astrocytes were incubated into 96-well plates at a density of about 1 × 10^4^ cells per well. Cell viability was estimated using 3-(4,5-dimethyl-2-thiazolyl)-2,5-diphenyl-2H-tetrazolium bromide (MTT; Solarbio) and lactate dehydrogenase (LDH) assay kit (Nanjing Jiancheng Bioengineering Institute, Nanjing, China). The optical absorbance was read on a plate reader at a wavelength of 490 nm for MTT. LDH release from damaged cell membrane was indicated as a percentage of total LDH according to the manufacturer’s instruction.

### Measurement of Superoxide Dismutase (SOD) Levels

The SOD activity in the astrocytes was measured by a commercially available kit (Nanjing Jiancheng Bioengineering Institute) according to the manufacturer’s instruction. Briefly, after treatment, cells were washed with cold PBS twice and collected. The homogenates were centrifuged for 10 min at 10,000 rpm at 4°C and supernatants were used for SOD activities. The optical absorbance was read on a plate reader at a wavelength of 450 nm. The protein concentration was determined by BCA assay.

### Intracellular ROS Assay

Astrocytes were seeded in 96-well plates at a density of 1 × 10^4^ cells per well. After exposure to OGD/R, the medium with different concentrations of TTB was replaced with 2′,7′-dichlorodihydrofluorescein diacetate (10 μM) in DMEM. The cells were incubated at 37°C for 30 min in the dark and then washed twice with PBS. The fluorescence was tested on a microplate reader using excitation/emission wavelengths (Ex/Em) of 488/525 nm.

### Western Blot Analysis

Total proteins were extracted with lysis radioimmunoprecipitation assay buffer (Applygen, Beijing, China) and protease inhibitor cocktail (Applygen). The protein concentrations were determined by BCA assay (Beyotime, Shanghai, China). All steps were carried out on ice. Nuclear and cytosolic proteins were extracted using a commercial kit (KeyGEN BioTECH’s, Nanjing, China). The extracts were boiled in a metal bath at 95°C for 5 min. Subsequently, sodium dodecyl sulfate-polyacrylamide gel electrophoresis was carried out to separate the proteins. The proteins were then transferred to a polyvinylidene fluoride (PVDF; Solarbio) membrane for about 1.5 h. After blocking in 5% nonfat milk (Applygen) for 2 h, the PVDF membrane was incubated with the primary antibodies anti-Nrf2 (1:500; Santa Cruz Biotechnology, Santa Cruz, CA, USA), anti-heme oxygenase-1 (HO-1; 1:500; Wanlei Biotechnology, Shenyang, China), anti-hypoxia-inducible factor-1α (HIF-1α; 1:500; BBI, Shanghai, China), anti-β-actin (1:5,000; abclonal, Wuhan, China), and anti-lamin B (1:500; abclonal) overnight at 4°C then followed by horseradish peroxidase-conjugated secondary antibodies for 2 h at room temperature. The membranes were washed three times for 10 min before obtaining protein bands by enhanced chemiluminescence reagents (Beyotime) and analyzed by ImageJ.

### Immunofluorescence Assay

The astrocytes were fixed with 4% paraformaldehyde for 30 min and permeabilized with 0.1% Triton X-100 (Solarbio) for 10 min at room temperature. After blocking with 3% bovine serum albumin for 30 min at room temperature, the cells were incubated with the primary antibody anti-Nrf2 (1:200; Santa Cruz Biotechnology) at 4°C overnight followed by Alexa Fluor 488 donkey anti-mouse antibody or Alexa Fluor 594 donkey anti-rabbit antibody (1:500; Invitrogen, Carlsbad, CA, USA). Nuclei were stained by 4′,6-diamidino-2-phenylindole (DAPI; 0.5 μg/ml; Beyotime), and images were acquired using a Zeiss fluorescence microscope attached to a digital camera.

### Real-Time Polymerase Chain Reaction (PCR)

Total RNA was extracted using Trizol reagent and dissolved in ultrapure distilled water (Invitrogen). Equal amounts of RNA were reverse transcribed at 25°C for 5 min, 42°C for 60 min, and 70°C for 5 min using RevertAid First Strand cDNA Synthesis Kit (Thermo Fisher Scientific, Waltham, MA, USA). cDNA amplification was carried out in 20 µl PCR buffer using AceQ quantitative PCR (qPCR) SYBR Green Master Mix (Vazyme, Nanjing, China). The primers used for amplification in the experiment were as follows: HIF-1α sense 5′-GTCTCCATTACCTGCCTCTG-3′ and antisense 5′-GATTCTTCGCTTCTGTGTCTTC-3′, vascular endothelial growth factor (VEGF) sense 5′-ACCCCACAAAGAGCTAGATAG-3′ and antisense 5′-CCTCTTCACTAAATGACAGTCCC-3′, and glial fibrillary acidic protein (GFAP) sense 5′-CCTTGCGCGGCACGAACGAG-3′ and antisense 5′-CCGAGCGAGTGCCTCCTGGT-3′. mRNA levels were normalized to levels of β-actin measured in the same samples (sense 5′-GCGTCCACCCGCGAGTACAA-3′ and antisense 5′-TCCATGGCGAACTGGTGGCG-3′).

### Astrocyte-Neuron Coculture

The astrocytes were passed in glass coverslips placed into 24-well plate at cell density of 1 × 10^5^ astrocytes per coverslip. When the cultures reached 70% to 80% confluence, the cells were subjected to OGD for 6 h and reoxygenation for 24 h; at the same time, primary neurons were extracted. After OGD/R in astrocytes was completed, primary neurons were seeded at a concentration of 1.2 × 10^4^ cells per well above the astrocytes and cocultured with DMEM for 24 h.

### Statistical Analysis

Statistical analyses were performed using Prism 5 software (GraphPad Software, Inc., San Diego, CA, USA). Data were expressed as the mean ± standard error (SE) of at least three independent experiments and compared using one-way analysis of variance with Tukey’s test. *p* < 0.05 was considered statistically significant difference.

## Results

### TTB Attenuated OGD/R-Induced Damage in Astrocytes

**Figure 1:**
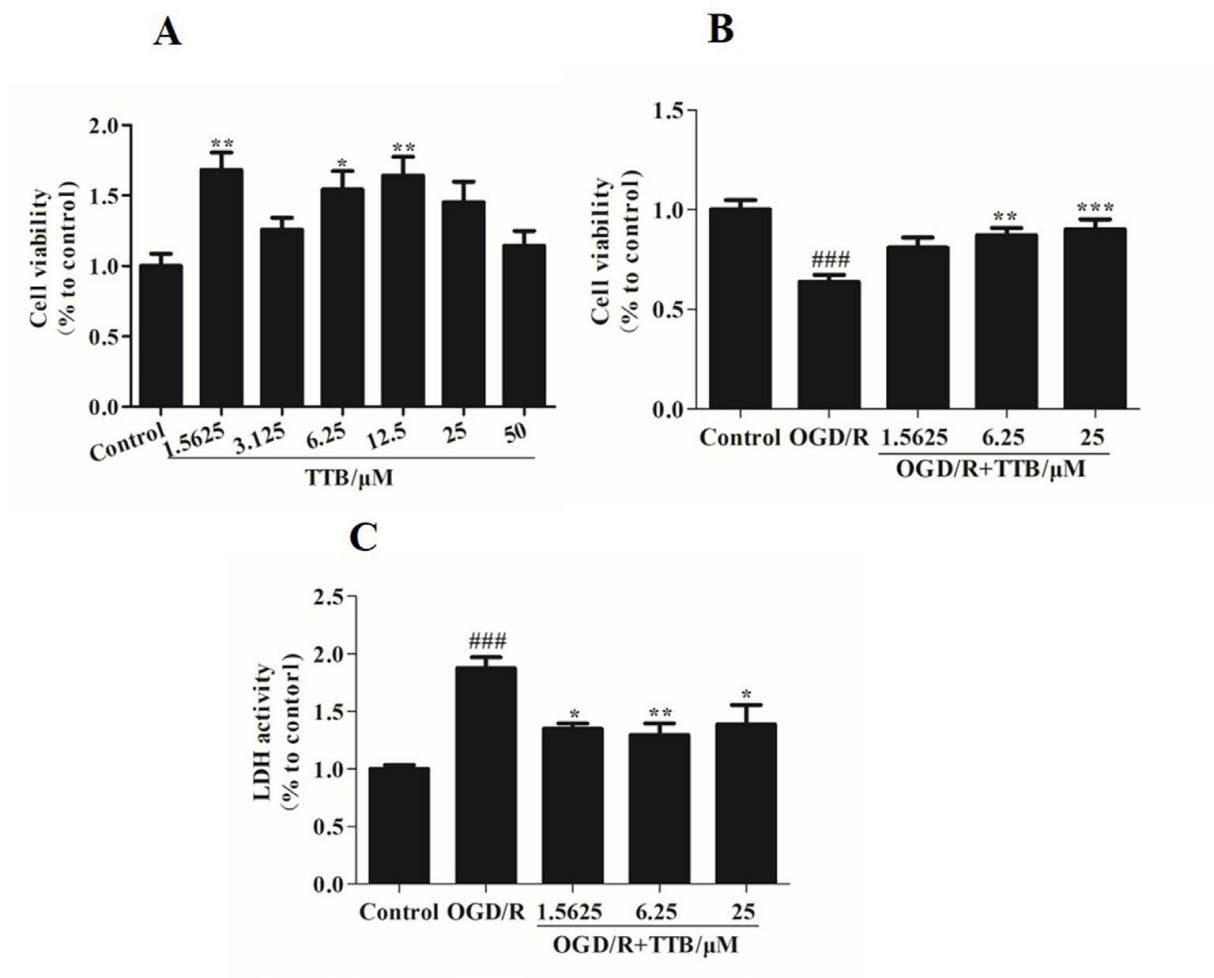
Cell toxicity of TTB in regular astrocytes and effect of TTB on cell viability under oxygen-glucose deprivation/reoxygenation (OGD/R) were evaluated. **(A)** Primary astrocytes were incubated with TTB at the concentration range from 1.5625 to 50 µM for 30 h in the normal incubator. Cell survival was estimated by the MTT assay. **(B** and **C)** Primary astrocytes were incubated with TTB at 1.5625, 6.25, and 25 µM for 6 h OGD and 24 h reoxygenation. Cell survival and cell death were estimated by the MTT and LDH assays, respectively. *p < 0.05, **p < 0.01 vs. blank control group; ^###^*p* < 0.001 vs. blank control group; *p < 0.05, **p < 0.01, ***p < 0.001 vs. OGD/R group. Data are mean ± SE of three independent experiments.

To examine the cell toxicity of TTB and the protective effect of TTB against cytotoxicity induced by OGD/R, the MTT assay was used to assess the viability of astrocytes. There was no cytotoxicity in the TTB concentration range from 1.5625 to 50 µM (**Figure 1A**). The viability of astrocytes exposed to OGD/R was significantly decreased compared to the blank control group, but this effect was reversed after treatment with TTB at concentrations of 1.5625, 6.25, and 25 µM (**Figure 1B**). These results indicated that TTB treatment was noncytotoxic and TTB attenuated OGD/R-induced astrocyte damage. Exposure to OGD/R significantly increased the release of LDH, whereas treatment with TTB markedly reduced the OGD/R-induced LDH release in astrocytes (**Figure 1C**).

### TTB Alleviated OGD/R-Induced Oxidative Stress in Astrocytes

**Figure 2:**
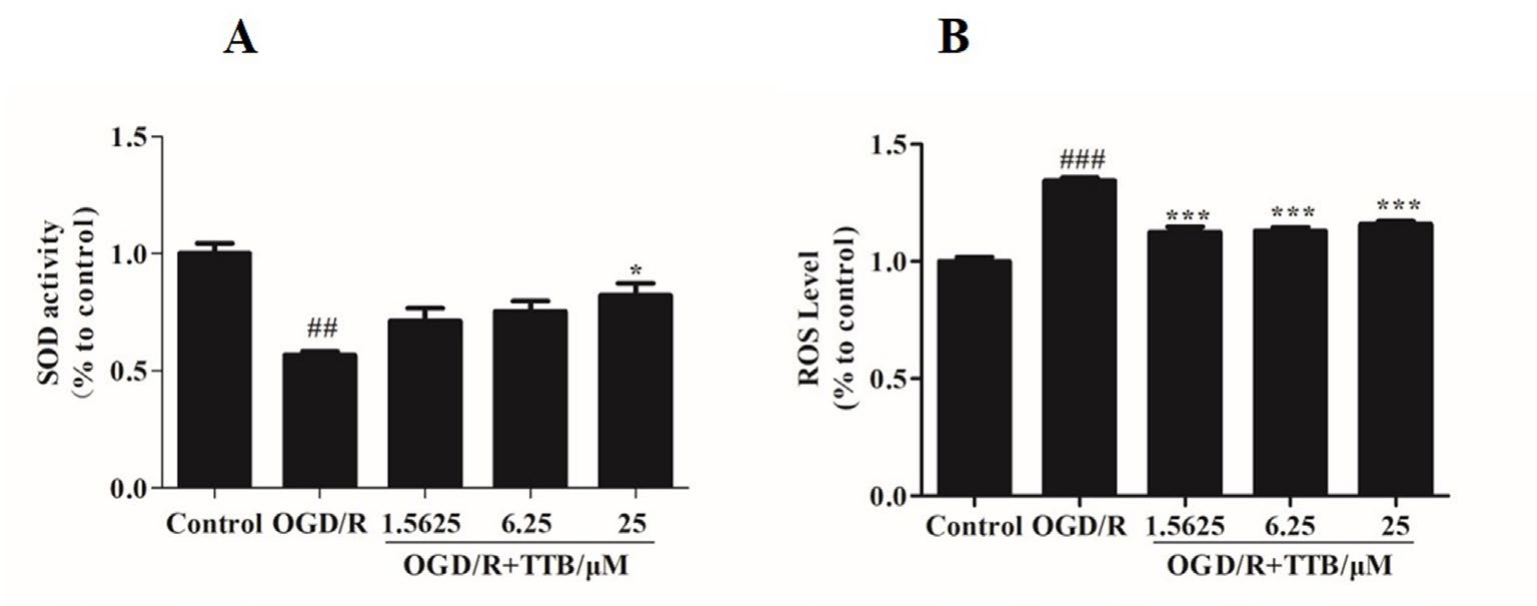
TTB inhibited the oxidative stress in astrocytes under OGD/R exposure. Primary astrocytes were treated with OGD for 6 h and reoxygenation for 24 h in the presence of TTB at 1.5625, 6.25, and 25 µM. **(A)** SOD activity was determined by the SOD assay. **(B)** ROS levels were estimated by the ROS assay. ^##^*p* < 0.01, ^###^*p* < 0.001 vs. blank control group; *p < 0.05, ***p < 0.001 vs. OGD/R group. Data are mean ± SE of three independent experiments.

To examine the effect of TTB treatment on OGD/R-induced oxidative stress in astrocytes, we examined the SOD activity and the ROS level. We found that treatment with TTB significantly reversed the decrease of SOD activity and the increase of intracellular ROS due to OGD/R (**Figures 2A, B**). These results indicated that TTB significantly improved OGD/R-induced oxidative stress in astrocytes.

### TTB Induced Nrf2 Up-Regulation and Nuclear Translocation in OGD/R-Injured Astrocytes

**Figure 3:**
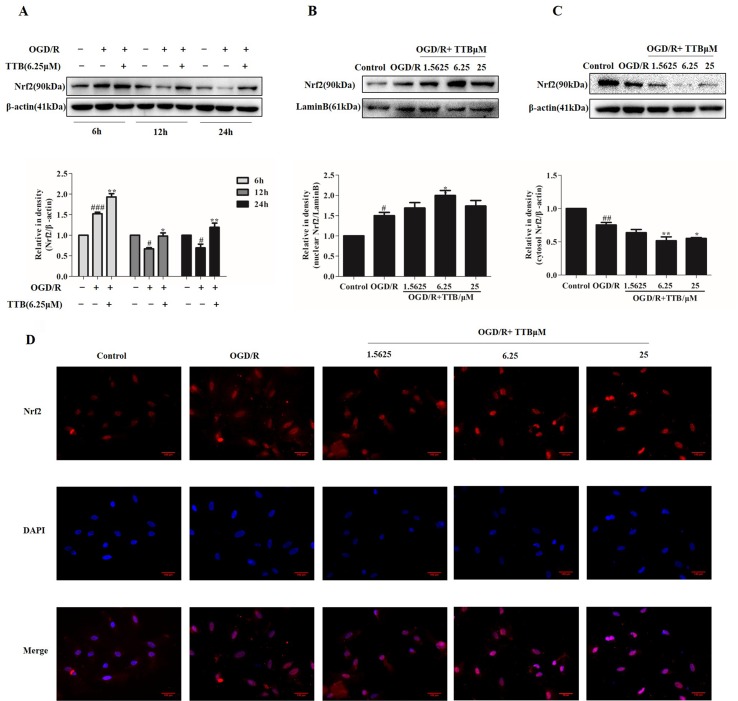
TTB induced Nrf2 activation and nuclear translocation in OGD/R-injured astrocytes. Primary astrocytes were incubated with TTB under OGD/R. Total proteins from treated astrocytes were extracted and used for Western blot. Nuclear and cytosolic proteins were determined by Western blot and immunofluorescence. **(A)** TTB at 6.25 µM increased Nrf2 expression in total protein under OGD for 6, 12, and 24 h after 24 h reoxygenation. **(B** and **C)** TTB at 1.5625, 6.25, and 25 µM decreased Nrf2 expression in the cytosol of astrocytes but increased in the nucleus at 6 h OGD and 24 h rexoygenation. Data are presented as relative density units normalized to β-actin. **(D)** Immunofluorescence staining was performed to detect the effects of TTB on Nrf2 translocation at 6 h OGD followed by 24 h rexoygenation. DAPI was used as a nuclei marker (40× magnification). ^#^ *p* < 0.05, ^##^ *p* < 0.01, ^###^ *p* < 0.001 vs. blank control group; *p < 0.05, **p < 0.01 vs. OGD/R group. Data are mean ± SE of three independent experiments.

When a stress response occurs, intracellular Nrf2 is easily transferred to the nucleus from the cytoplasm, which subsequently initiates the transcriptional activation of various antioxidant enzymes and phase II detoxification enzymes. The effect of TTB on Nrf2 expression and nuclear translocation in OGD/R-induced astrocytes was determined by Western blot and immunofluorescence. Lamin B was used to assess the purity of the nuclear fraction. As shown in **Figure 3A**, compared to the control group, Nrf2 protein expression was dramatically increased by OGD for 6 h and reoxygenation for 24 h but decreased by 12 and 24 h OGD and 24 h reoxygenation. TTB at 6.25 µM up-regulated Nrf2 protein expression in all three time courses under OGD/R. Moreover, OGD for 6 h and reoxygenation for 24 h significantly increased Nrf2 nuclear translocation in astrocytes, and treatment with TTB at 1.5625, 6.25, and 25 µM further facilitated Nrf2 translocation to the nucleus compared to the OGD/R group (**Figures 3B–D**). These results indicated that TTB treatment could up-regulate Nrf2 expression and promote Nrf2 nuclear translocation under OGD/R condition.

### TTB Activates the Nrf2/HO-1 Pathway

**Figure 4:**
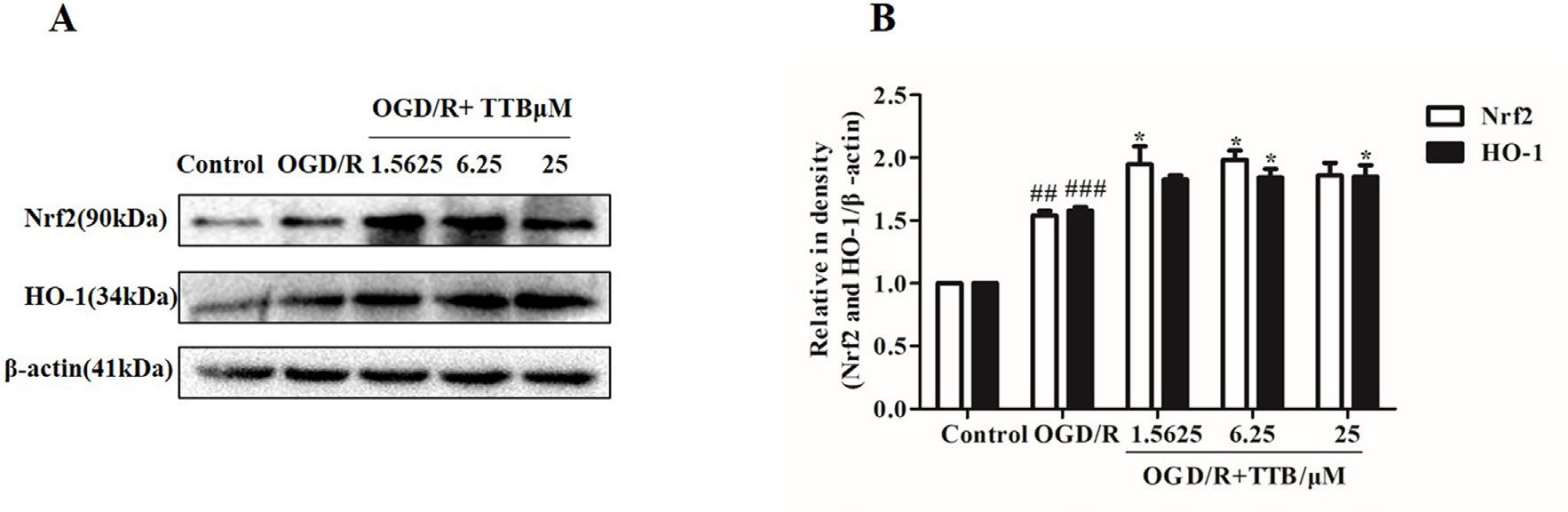
TTB treatment activated Nrf2/HO-1 expression. Primary astrocytes were incubated with TTB at 1.5625, 6.25, and 25 µM for 6 h OGD and 24 h reoxygenation. **(A** and **B)** Western blot was carried out to determine the expression of Nrf2 and HO-1, respectively. Data are presented as relative density units normalized to β-actin ^##^ *p* < 0.01, ^###^*p* < 0.001 vs. blank control group; *p < 0.05 vs. OGD/R group. Data are mean ± SE of three independent experiments.

Nrf2 is activated under stress conditions and translocates to the nucleus to initiate transcriptional activation of HO-1. Therefore, we examined the effect of TTB on the expression of Nrf2 and HO-1 proteins in astrocytes by Western blot. Cells were incubated with TTB at 1.5625, 6.25, and 25 µM under OGD for 6 h and reoxygenation for 24 h. As a result, we found that, compared to the OGD/R group, Nrf2 and HO-1 expression in protein level was significantly up-regulated in the OGD/R+TTB group (**Figures 4A, B**), which indicated that TTB might get involved in the Nrf2/HO-1 signal pathway.

### TTB Inhibited OGD-Induced HIF-1α Accumulation, VEGF Release, and GFAP Expression in Astrocytes

**Figure 5:**
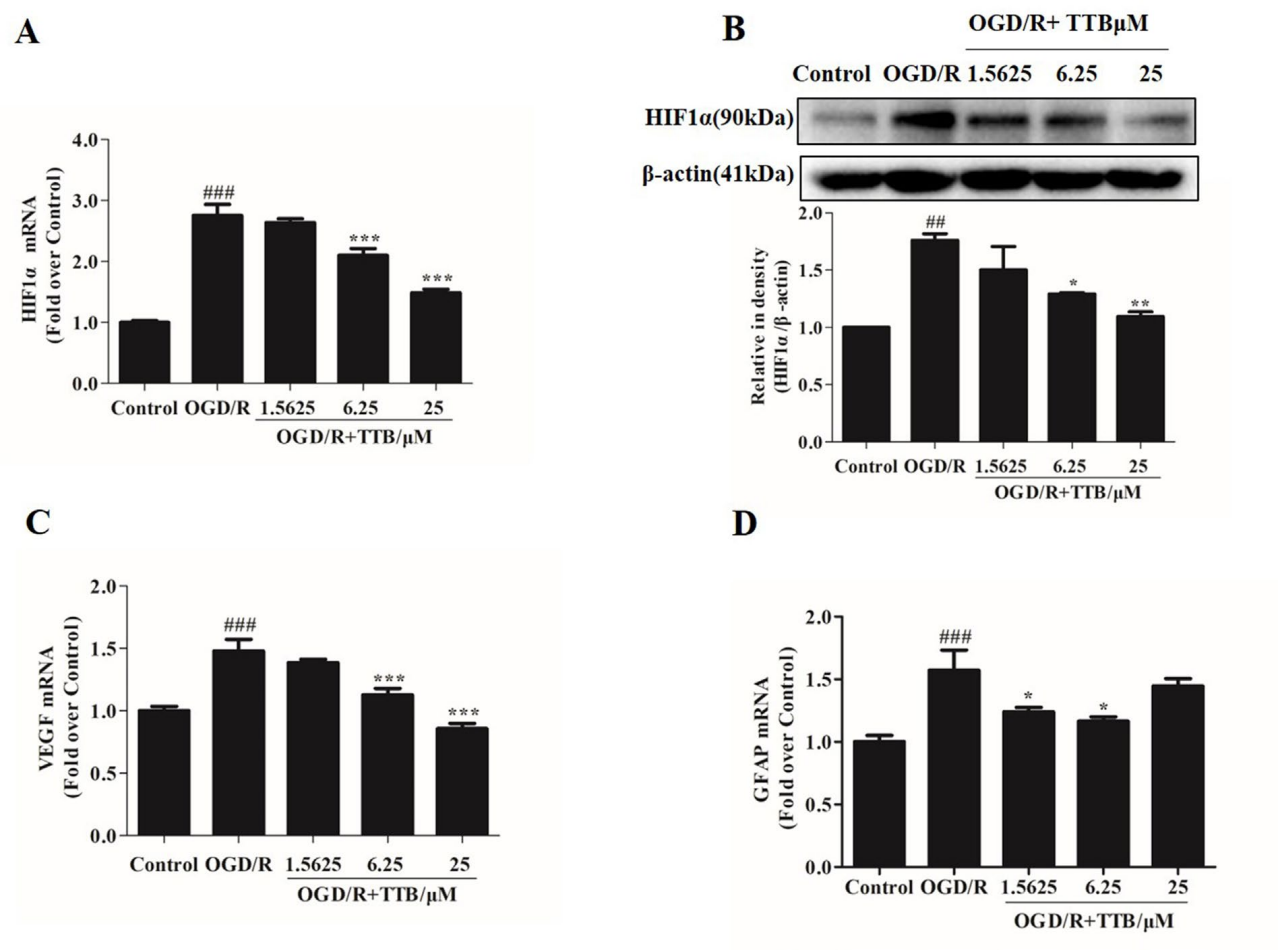
TTB reduced HIF-1α, VEGF, and GFAP expression in OGD/R-injured astrocytes. Primary astrocytes were incubated with TTB at 1.5625, 6.25, and 25 µM for 6 h OGD and 24 h reoxygenation. RNA was extracted and HIF-1α, VEGF, and GFAP mRNA levels were quantified by qPCR. Western blot was carried out for HIF-1α protein determination. Results were normalized to β-actin and expressed as fold over control. **(A** and **B)** HIF-1α mRNA expression was reduced by TTB treatment in OGD/R-injured astrocytes, which was consistent with HIF-1α protein level. **(C** and **D)** TTB inhibited VEGF and GFAP mRNA expression in OGD/R-injured astrocytes. ^##^*p* < 0.01, ^###^*p* < 0.001 vs. blank control group; *p < 0.05, **p < 0.01, ***p < 0.001 vs. OGD/R group. Data are mean ± SE of three independent experiments.

OGD/R injury could trigger HIF-1α up-regulation. Nrf2 and HIF-1α are two transcription factors that represent oxygen and redox state. We further observed the effect of TTB on HIF-1α expression in both protein and mRNA levels under OGD/R condition. Cells were treated with TTB in different concentrations (1.5625, 6.25, and 25 µM) for 6 h OGD and 24 h reoxygenation in astrocytes. The results showed that TTB treatment inhibited OGD/R-induced up-regulation of HIF-1α (**Figures 5A, B**). Moreover, VEGF and GFAP gene expressions were measured by qPCR. TTB inhibited OGD/R-induced increase in GFAP and VEGF gene expressions (**Figures 5C, D**).

### TTB Prevented OGD/R-Induced Inhibition of Neurite Outgrowth in Neuron-Astrocyte Coculture System

**Figure 6:**
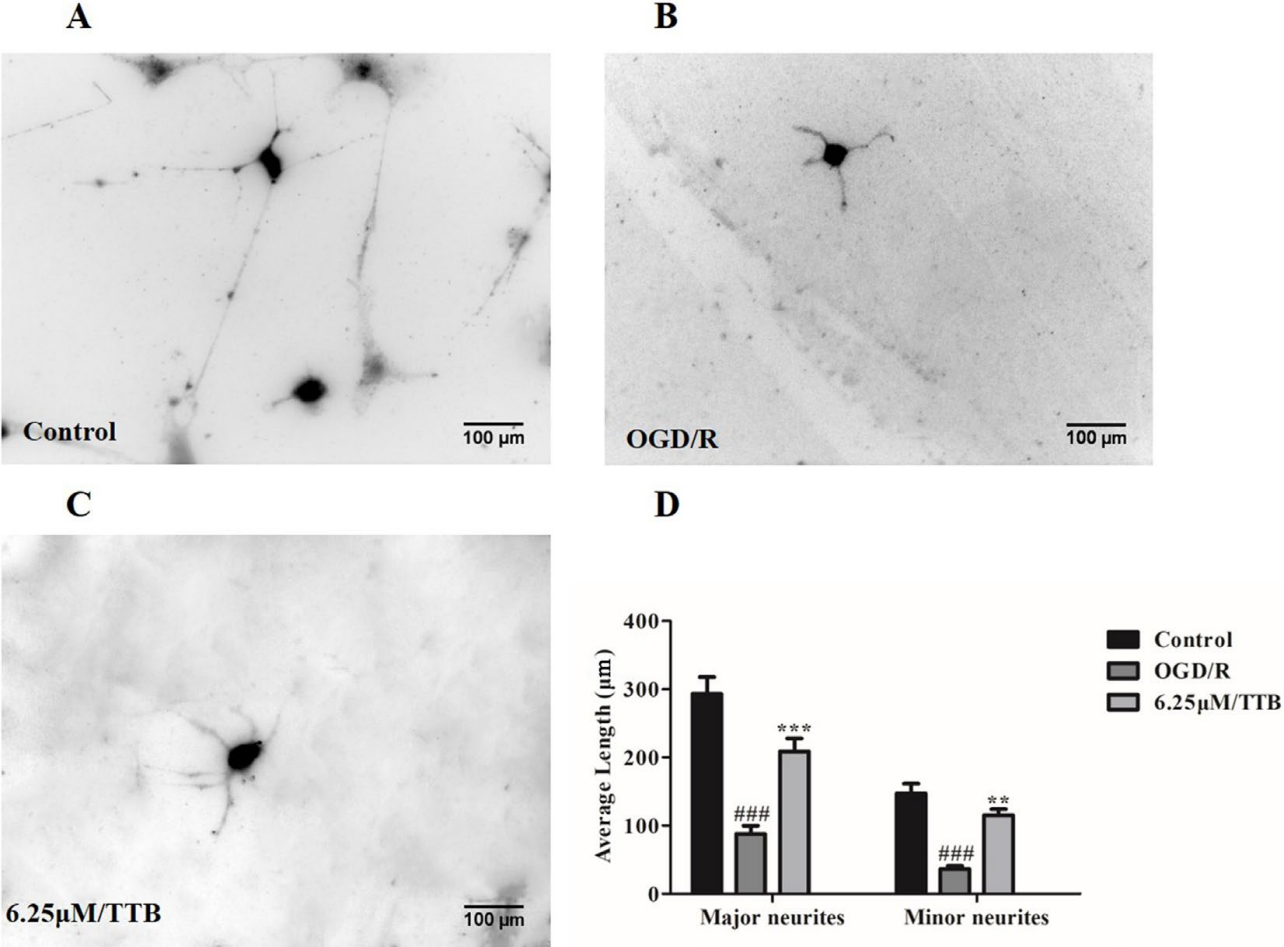
Effects of TTB-treated astrocytes on hippocampal neuron neurite outgrowth. Astrocytes were treated with 6.25 µM TTB for 6 h OGD and 24 h reoxygenation. Hippocampal neurons were plated on top of pretreated astrocytes for an additional 24 h. Then, cultures were fixed and stained with the antibody of neuron-specific β-III-tubulin and a fluorescent secondary antibody. ImageJ was used to measure neurite length. **(A)** Control, **(B)** OGD/R, **(C)** TTB treatment, and **(D)** morphometric quantification of major neurite and minor neurite length. ^###^*p* < 0.001 vs. blank control group; **p < 0.01, ***p < 0.001 vs. OGD/R group. Data are mean ± SE of three independent experiments.

To test the hypothesis that OGD-treated astrocytes inhibit neurite outgrowth and whether TTB could alleviate the inhibition, we plated the neurons on top of the OGD/R-treated astrocytes in the presence of TTB at a concentration of 6.25 µM. We observed that neurons cocultured with OGD/R-induced astrocytes developed shorter major and minor neurites compared to neurons cocultured with the control astrocytes, whereas TTB attenuated the OGD/R-induce inhibition of neurite growth. (**Figure 6A–D**). This result indicated that TTB could regulate astrocyte function and promote neuronal growth.

### Nrf2 Inhibitor Abolished the Protective Effect of TTB by the Nrf2/HO-1 Pathway in OGD/R-Injured Astrocytes

**Figure 7:**
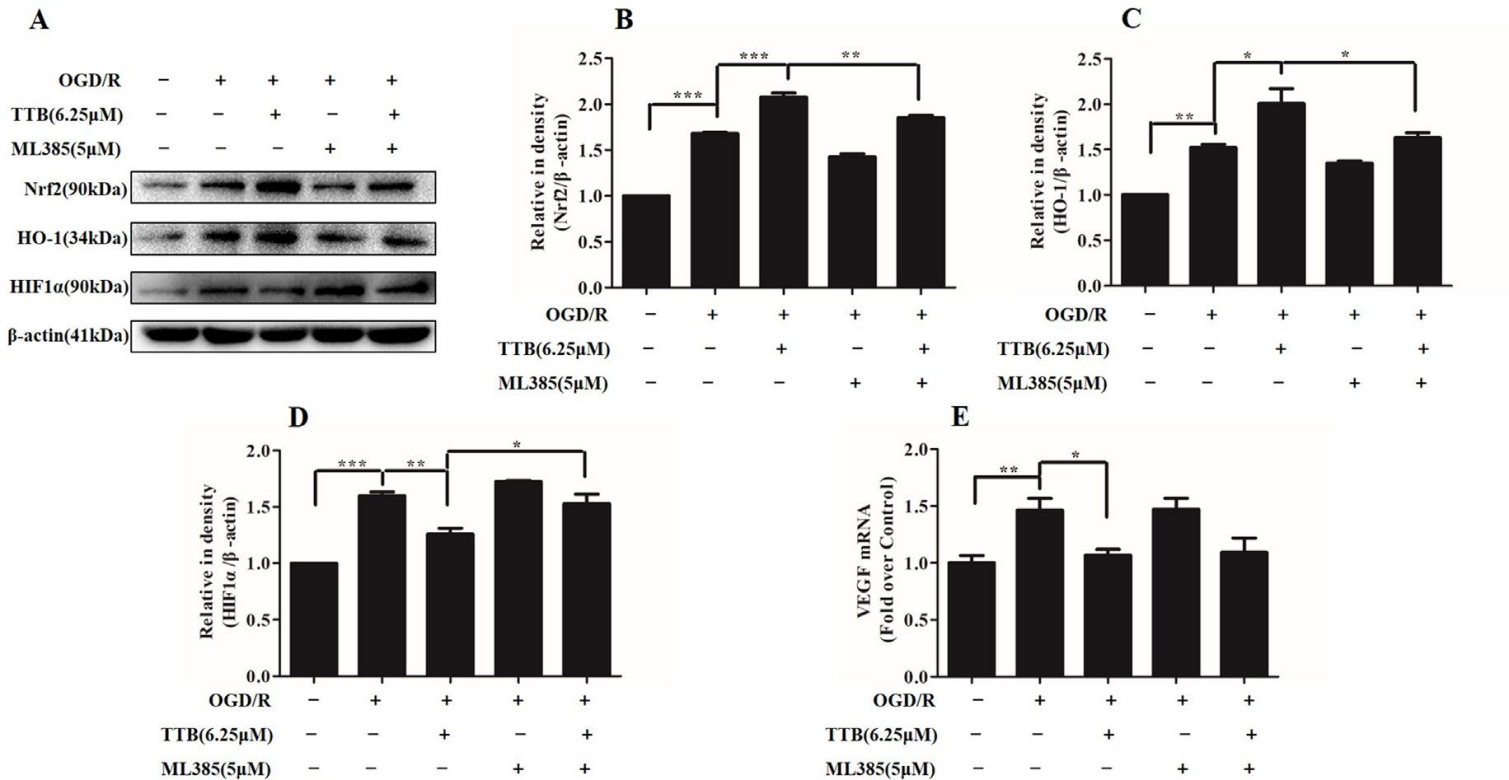
Effects of ML385 (Nrf2-specific inhibitor) on Nrf2, HO-1, HIF-1α, and VEGF levels in OGD/R-treated astrocytes. Western blot and qPCR were carried out. Astrocytes were preincubated with ML385 at a concentration of 5 µM for 12 h and then cells were treated with 6.25 µM TTB for 6 h OGD and 24 h reoxygenation. **(A–D)** Expression of Nrf2, HO-1, and HIF-1α was determined by Western blot, respectively. **(E)** Expression of VEGF in the mRNA level was determined by qPCR. *p < 0.05, **p < 0.01, ***p < 0.001. Data are mean ± SE of three independent experiments.

To investigate whether Nrf2 function contributes to the neuroprotective effects of TTB, astrocytes were incubated with ML385, the Nrf2-specific inhibitor, to inhibit Nrf2 expression. Under OGD/R treatment, the expression of Nrf2 and HO-1 was inhibited by ML385 at a concentration of 5 µM. ML385 also abolished TTB-induced increase in Nrf2 and HO-1 expression (**Figures 7A–C**). Moreover, the effect of TTB on the expression of HIF-1α and VEGF was inhibited by ML385 (**Figures 7A, D, E**). These data demonstrated that the neuroprotective effect of TTB may be through the activation of the Nrf2/HO-1 pathway.

## Discussion

NHIE causes a series of oxidative bursts, cell apoptosis, and cascade of inflammatory responses. The potential therapy strategies have been limited and unsatisfactory (Mulkey et al., 2011; Zalewska et al., 2015). Astrocyte dysfunction is critically involved in oxidative stress, apoptosis, and inflammation in the pathologic process of NHIE. The current studies were undertaken to identify the hypothesis that TTB exposure would initiate the protective response against OGD/R-induced injury in astrocytes, which is an *in vitro* model to mimic NHIE. The transcription factor Nrf2 was identified to play an important role in modulating the neuroprotective effects of TTB.

TTB is a natural biphenanthrene that is a relatively rare secondary metabolite in the plant kingdom. TTB was only reported to have cytotoxicity against HGC-27 and HT-29 cancer cell lines (Liu et al., 2016b). TTB contains phenolic hydroxyl groups, which determines its significant antioxidant activity (Quiniou et al., 2008). Recent studies have identified that antioxidants can protect astrocytes from hypoxia/ischemia (H/I)-induced dysfunction (Bao et al., 2016). In the present study, we exposed primary cultures of astrocytes to OGD for 6 h and reoxygenation for 24 h. Our results demonstrated for the first time that TTB at 6.25 and 25 μM increased cell survival significantly in OGD/R-induced injury. TTB also decreased the LDH release to the culture medium.

H/I exposure causes an oxidative stress and induces a significant damage in brain tissue, which can be described as an increase in the rate of ROS generation and imbalance of antioxidant defense system in the molecular level (Brekke et al., 2017; Parfenova et al., 2018). Excessive ROS initiates pro-inflammatory or growth stimulatory signals that are associated with cell death. Therefore, new pharmacological strategies aimed at the antioxidant system may potentially improve clinical management. The present study demonstrated that OGD/R exposure markedly increased ROS production in astrocytes compared to the control group and this increase was attenuated by TTB treatment. SODs, the antioxidant enzymes, are generally considered as O^2-^ scavengers against tissue and cellular damage caused by ROS (Fridovich, 1995; Li et al., 2008). In our study, OGD/R induced the decrease of SOD activity and TTB prevented the decrease of SOD activity in response to OGD/R. These data illustrated that TTB treatment had protective roles against OGD/R-induced oxidative stress.

Nrf2 is a well-known key regulator of cellular resistance to oxidants and is activated through translocation from the cytoplasm to the nucleus, where it induces HO-1 gene expression as a target antioxidative gene (Kensler et al., 2007; Wu et al., 2015). HO-1, a rate-limiting enzyme in the transition of heme into biliverdin, also has a pivotal function in response to oxidative stress (Cao et al., 2017). When Nrf2 is up-regulated and translocated into the nucleus from the cytoplasm under stress conditions, the process is also essential for the activation of HO-1 expression (Kang et al., 2015). Growing evidence demonstrated that the Nrf2/HO-1 signaling pathway participated in the process of oxidative stress in several brain dysfunctional diseases (Meng et al., 2016; Bellaver et al., 2017; Zhao et al., 2018). Several antioxidant ingredients indicated that they protected cell damage by up-regulating Nrf2 and HO-1 expression in various diseases (Hsu et al., 2012; Yao et al., 2015; Jung et al., 2017). To explore whether TTB-induced cytoprotection was dependent on the presence of Nrf2 by inhibiting oxidative stress, the astrocytes were treated with OGD in different time courses of 6, 12, and 24 h followed by reoxygenation for 24 h. Our results showed that the protein levels of Nrf2 were increased by OGD for 6 h and reoxygenation for 24 h but decreased by OGD for 12 or 24 h and reoxygenation for 24 h compared to the control group. Meanwhile, TTB up-regulated Nrf2 expression in total protein at all time points compared to the OGD/R group. The results demonstrated TTB potentially prolonged the activation state of the Nrf2 pathway and maintained the protection against oxidative stress in OGD/R-induced astrocytes. Furthermore, with OGD for 6 h and reoxygenation for 24 h treatment, TTB facilitated Nrf2 translocation to the nucleus and increased Nrf2 expression in the nucleus, suggesting that TTB promoted the activation of the Nrf2/HO-1 pathway in H/I injury in the early phase.

HIF-1α is an important transcription factor in a wide variety of responses to hypoxia (Chavez et al., 2000). Using the astrocyte-neuron coculture model, the selective loss of HIF-1α function in neuron induced neuronal susceptibility to H/I injury, whereas the loss of HIF-1α function in astrocytes inhibits neuronal death by hypoxia (Vangeison et al., 2008). During hypoxia-induced CNS injury, HIF-1α expression targets multiple genes, including VEGF. The activation of VEGF expression under hypoxic conditions has been investigated in several studies. Notably, astrocytes secrete basal levels of VEGF under physiological conditions and the expression is further up-regulated by hypoxia. VEGF gene expression is transcriptionally regulated by HIF-1α (Marti et al., 2000; Schmid-Brunclik et al., 2008; Wiesner et al., 2013). Some previous studies identified that VEGF protects neurons from ischemic insults and promoted neurogenesis after cerebral ischemic injury (Ma et al., 2012; Liu et al., 2018). However, other studies reported that anti-VEGF treatment blocks vascular leakage in hypoxia (Nordal et al., 2004; Kaur et al., 2006). In the present study, it showed that OGD/R induced HIF-1α and VEGF up-regulation. TTB inverted the effect of OGD/R on HIF-1α/VEGF expression in astrocytes. The results disclosed that the HIF-1α/VEGF pathway might be involved in the astrocyte oxidative stress, providing new insights into TTB protection.

Nrf2 and HIF-1α represent the oxygen and redox state-dependent transcription factors. Their stabilization by redox status decides the cell fate, which means the existence of interplay between Nrf2 and the HIF-1α/VEGF signaling pathway under H/I injury. One study demonstrated that hypoxia induced Nrf2 activation, resulting in the induction of Nrf2-dependent target thioredoxin-1 enhancement of HIF-1α response in A549 cells (Malec et al., 2010). Li et al. indicated that Nrf2 knockdown inhibits venous hypertension-induced activation of the HIF-1α/VEGF pathway (Li et al., 2016). In our study, TTB may act as an Nrf2 activator that up-regulated and maintained Nrf2 expression after OGD/R. To further explore whether the protection of TTB on OGD/R-induced injury in astrocytes was dependent on the activation of the Nrf2 pathway, ML385, a small-molecule Nrf2 inhibitor, was implemented to observe the protective mechanism of TTB. ML385 increases the ubiquitination and inhibits the proteasome degradation of Nrf2 binding to Keap1 and subsequently suppresses Nrf2 expression (Jung et al., 2018). Our results displayed that, with the combination of TTB and MLB385 treatment, MLB385 reversed the TTB-induced up-regulation of Nrf2 and HO-1 expression in OGD/R-induced astrocytes. These results indicated that the activation of the Nrf2/HO-1 signaling pathway after TTB treatment was responsible for the protection of antioxidative stress. Furthermore, we found that, under OGD/R treatment in astrocytes, MLB385 induced the maintenance of the high level of HIF-1α expression. The combination of TTB and MLB385 decreased the HIF-1α protein level compared to MLB385 alone. These results suggested that TTB inhibited OGD/R-induced astrocyte oxidative stress at least partially through the down-regulation of HIF-1α and VEGF *via* the Nrf2/HO-1 signaling pathway.

CNS diseases, such as trauma, H/I injury, neuroinflammation, or neurodegeneration, cause astrocytes to become reactive. Reactive astrocytes were verified to control formation, maintenance, function, and the removal of neuronal synapses (Eroglu and Barres, 2010; Koizumi et al., 2018). Our study verified that OGD/R induced astrocyte reactivation by up-regulating GFAP expression. Meanwhile, TTB inhibited GFAP expression, which revealed that TTB inhibited OGD/R-induced astrocyte reactivation. The mechanism might go through the alteration of factor secretion and gene expression. A previous study showed that proteins released by astrocytes selectively increased neuron axon length, branching, function, and synapse formation (Hughes et al., 2010). Other study demonstrated that astrocytes produced mRNAs that encoded synaptic adhesion proteins, which affected neuronal synapse formation (Cahoy et al., 2008). Astrocyte-neuron interaction might participate in neuronal plasticity. In our study, with neuron-astrocyte coculture, OGD/R-induced astrocytes inhibited neurite growth in neurons compared to the control group. TTB-treated astrocytes reversed the inhibition of OGD/R on neurite growth of neurons in the coculture system. It suggested that TTB regulated astrocyte function and subsequently promoted neuronal plasticity under H/I injury. However, the deep mechanism of which factors were secreted by astrocytes and which genes were regulated remains unknown.

Taken together, TTB displays antioxidant activities in OGD/R-induced astrocytes. Our study provides evidence that TTB effectively suppresses excessive ROS production and increases SOD activity in terms of attenuation of HIF-1α and VEGF expression by activating the Nrf2/HO-1 pathway, which depends on Nrf2 nuclear translocation and up-regulation of HO-1, to protect OGD/R-induced cell oxidative stress. Also, TTB administration in reactive astrocytes by OGD/R might contribute to reverse the inhibition of OGD/R on neurite growth in neurons. These data suggest that TTB could be a novel medication that imparts effective neuroprotection against NHIE to prevent cerebral oxidative stress-induced injury.

## Data Availability

The datasets for this manuscript are not publicly available because all the data can be found in the manuscript. Requests to access the datasets should be directed to enjoyyz@163.com.

## Ethics Statement

This study was carried out in accordance with the recommendations of the Medical College of Yangzhou University Guide for Care and Use of Laboratory Animals. The protocol was approved by the Committee of Care and Use of Laboratory of Medical College of Yangzhou University.

## Author Contributions

XZ and LL contributed to the design of the study. LL, ZZ, QY, and XZ performed the experiments. ZZ, LL, and XZ analyzed and interpreted the data. XZ, LL, and ZZ drafted and revised the manuscript. All the authors approved the final version of manuscript.

## Funding

This study was supported by the National Natural Science Foundation of China (81701211 and 81703812), Traditional Chinese Medicine Bureau of Jiangsu Province Project (YB2015182), Jiangsu Pharmaceutical Association-Aosaikang Clinical Pharmacy Foundation (A201737), Qinglan Project of Yangzhou University (20180210), and “Summit of the Six Top Talents” Program of Jiangsu Province (WSN-277).

## Conflict of Interest Statement

The authors declare that the research was conducted in the absence of any commercial or financial relationships that could be construed as a potential conflict of interest.

## Abbreviations

NHIE, neonatal hypoxic/ischemic encephalopathy; TTB, 2,7,2′-trihydroxy-4,4′7′-trimethoxy-1,1′-biphenanthrene; OGD/R, oxygen-glucose deprivation/reoxygenation; CNS, central nervous system; ROS, reactive oxygen species; SOD, superoxide dismutase; LDH, lactate dehydrogenase; Nrf2, nuclear factor erythroid 2-related factor 2; HO-1, heme oxygenase-1; HIF-1α, hypoxia-inducible factor-1α; VEGF, vascular endothelial growth factor; GFAP, glial fibrillary acidic protein; Keap1, Kelch-like ECH-associated protein 1; DMEM, high-glucose Dulbecco’s modified Eagle medium; MTT, 3-(4,5-dimethyl-2-thiazolyl)-2,5-diphenyl-2H-tetrazolium bromide; H/I, hypoxia/ischemia.
